# CME ACTIVITY: Microbial Interactions during Upper Respiratory Tract Infections

**DOI:** 10.3201/eid1410.070616

**Published:** 2008-10

Medscape, LLC is pleased to provide online continuing medical education (CME) for this journal article, allowing clinicians the opportunity to earn CME credit. Medscape, LLC is accredited by the Accreditation Council for Continuing Medical Education (ACCME) to provide CME for physicians. Medscape, LLC designates this educational activity for a maximum of 0.5 *AMA PRA Category 1 Credits*™. Physicians should only claim credit commensurate with the extent of their participation in the activity. All other clinicians completing this activity will be issued a certificate of participation. To participate in this journal CME activity: (1) review the learning objectives and author disclosures; (2) study the education content; (3) take the post-test and/or complete the evaluation at **http://www.medscape.com/cme/eid**; (4) view/print certificate.

## Learning Objectives

Upon completion of this activity, participants will be able to:

Identify common bacterial isolates from children with upper respiratory infection
Specify significant interactions between colonizing bacteria during upper respiratory infection
Identify variables associated with higher rates of colonization with *Streptococcus pneumoniae*
Specify which bacteria is more common in the nasopharynx of children who attend day care

## EDITOR

**Beverly Merritt,** Technical Writer-Editor, Emerging Infectious Diseases. *Disclosure: Beverly Merritt has disclosed no relevant financial relationships.*

## CME AUTHOR

**Charles P. Vega, MD**, Associate Professor; Residency Director, Department of Family Medicine, University of California, Irvine. *Disclosure: Charles P. Vega, MD, has disclosed that he has served as an advisor or consultant to Novartis, Inc.*

## AUTHORS

Disclosures: **Melinda M. Pettigrew, PhD**; **Janneane F. Gent, PhD; Krystal Revai, MD; Janak A. Patel, MD; and Tasnee Chonmaitree, MD,** have disclosed no relevant financial relationships.

## Earning CME Credit

To obtain credit, you should first read the journal article. After reading the article, you should be able to answer the following, related, multiple-choice questions. To complete the questions and earn continuing medical education (CME) credit, please go to **http://www.medscape.com/cme/eid**. Credit cannot be obtained for tests completed on paper, although you may use the worksheet below to keep a record of your answers. You must be a registered user on Medscape.com. If you are not registered on Medscape.com, please click on the New Users: Free Registration link on the left hand side of the website to register. Only one answer is correct for each question. Once you successfully answer all post-test questions you will be able to view and/or print your certificate. For questions regarding the content of this activity, contact the accredited provider, CME@medscape.net. For technical assistance, contact CME@webmd.net. American Medical Association’s Physician’s Recognition Award (AMA PRA) credits are accepted in the US as evidence of participation in CME activities. For further information on this award, please refer to http://www.ama-assn.org/ama/pub/category/2922.html. The AMA has determined that physicians not licensed in the US who participate in this CME activity are eligible for *AMA PRA Category 1 Credits*™. Through agreements that the AMA has made with agencies in some countries, AMA PRA credit is acceptable as evidence of participation in CME activities. If you are not licensed in the US and want to obtain an AMA PRA CME credit, please complete the questions online, print the certificate and present it to your national medical association.

### Article Title: Microbial Interactions during Upper Respiratory Tract Infections

## CME Questions

1. Which of the following bacteria was most commonly isolated from nasopharyngeal swabs in the current study?
  A. *Staphylococcus aureus*
  B. *Moraxella catarrhalis*
  C. *Streptococcus pneumoniae*
  D. *Haemophilus influenzae*
2. Which of the following associations between bacteria in the current study is most accurate?
  A. Colonization with *H. influenzae* was positively associated with *S. pneumoniae* colonization
  B. Colonization with *M. catarrhalis* was positively associated with *S. pneumoniae* colonization
  C. Colonization with *S. pneumoniae* was positively associated with *M. catarrhalis* colonization
  D. Colonization with *H. influenzae* and *M. catarrhalis* was positively associated with *S. pneumoniae* colonization
3. Which of the following variables was associated with a significant decrease in the rate of colonization with *S. pneumoniae*?
  A. Antibiotic use in the past 7 days
  B. Younger age
  C. Up-to-date vaccination with pneumococcal vaccine (PCV7)
  D. Breast-feeding
4. Day care promoted colonization with which of the following bacteria?
  A. *S. aureus*
  B. *M. catarrhalis*
  C. *S. pneumoniae*
  D. *H. influenzae*

### Activity Evaluation

**Table:** 

**1. The activity supported the learning objectives.**				
Strongly Disagree				Strongly Agree
1	2	3	4	5
**2. The material was organized clearly for learning to occur.**				
Strongly Disagree				Strongly Agree
1	2	3	4	5
**3. The content learned from this activity will impact my practice.**				
Strongly Disagree				Strongly Agree
1	2	3	4	5
**4. The activity was presented objectively and free of commercial bias.**				
Strongly Disagree				Strongly Agree
1	2	3	4	5

